# Supplementary material for: Genomic and Metabolic Insights into Two Novel Thiothrix Species from Enhanced Biological Phosphorus Removal Systems
Source: Microorganisms. 2020 Dec 18;8(12):2030. doi: 10.3390/microorganisms8122030 (PMC7767063; doi:10.3390/microorganisms8122030)
Supplement: Supplementary file 1 [file microorganisms-08-02030-s001.pdf]

Table S1. Identity level of 16S rRNA genes in representatives of the genus *Thiothrix*,%.

	Thiothrix sp. RT	*Thiothrix* sp.SSD2	*T. nivea* DSM 5205^T	*T.unzii* A1^T	*T. lacustris* BL^T	*T. caldifontis* G1^T	*T. fructosivorans* Q^T
Thiothrix sp RT	100.0	93.0	97.9	95.9	96.1	96.3	94.9
Thiothrix sp SSD2	94.0	100.0	96.1	94.8	94.6	94.3	93.3
T. nivea DSM 5205^T	97.9	96.1	100.0	94.6	95.1	95.1	94.3
T. unzii A1^T	95.9	94.8	95.41	100.0	95.4	95.5	95.7
T. lacustris BL^T	96.1	94.6	94.6	94.8	100.0	98.7	98.6
T. caldifontis G1^T	96.3	94.3	95.1	95.5	98.7	100.0	98.9

Table S2. Molecular characteristics of representatives of the genus *Thiothrix*.

Species	*T. nivea* DSM 5205^T	*T. lacustris* BL^T	*T. caldifontis* G1^T	*Thiothrix* sp. RT	*Thiothrix* sp. SSD2
**% Average nucleotide identity (ANI) to:**					
T. nivea DSM 5205^T	100.0	76.7	75.5	77.5	80.2
T. lacustris BL^T	76.7	100.0	79.0	76.5	75.9
T. caldifontis G1^T	75.5	79.0	100.0	76.7	74.8
Thiothrix sp. RT	77.5	76.5	76.7	100.0	76.7
Thiothrix sp. SSD2	80.2	75.9	74.8	76.7	100.0
**% dDDH to:**					
T. nivea DSM 5205^T	100.0	23.9	23.8	25.6	27.3
T. lacustris BL^T	23.9	100.0	28.0	24.9	22.4
T. caldifontis G1^T	23.8	28.0	100.0	30.2	22.7
Thiothrix sp. RT	25.6	24.9	30.2	100.0	24.9
Thiothrix sp. SSD2	27.3	22.4	22.7	24.9	100.0

Table S3. Genes encoding enzymes of metabolic pathways in *Thiothrix* sp. SSD2 and *Thiothrix* sp. RT.

Gene	Predicted function	*Thiothrix* sp. RT	*Thiothrix* sp. SSD2
		RefSeq (GeneBank) ID of gene	RefSeq (GeneBank) ID of gene
**Sulfur metabolism**			
sqrA	Sulfide quinone oxidoreductase SqrA (EC 1.8.5.4)	fig|6666666.654935.peg.2381	QLQ30307.1
sqrF	Sulfide quinone oxidoreductase SqrF (EC 1.8.5.4)	fig|6666666.654935.peg.1874	QLQ32374.1
fccB	Sulfide dehydrogenase (flavocytochrome c) flavoprotein chain	fig|6666666.654935.peg.278<br>fig|6666666.654935.peg.268<br>fig|6666666.654935.peg.1885<br>fig|6666666.654935.peg.1391	QLQ32594.1
fccA	cytochrome c subunit of flavocytochrome c sulfide dehydrogenase	fig|6666666.654935.peg.269	QLQ32595.1

sqrD	Sulfide quinone oxidoreductase SqrD (EC 1.8.5.4)	—	QLQ32934.1
soxA	sulfur oxidation c-type cytochrome SoxA (EC 1.8.2.3)	fig|6666666.654935.peg.3301<br>fig|6666666.654935.peg.708	QLQ32948.1<br>QLQ33792.1
soxZ	thiosulfate oxidation carrier complex protein SoxZ (EC 1.8.2.6)	fig|6666666.654935.peg.2874<br>fig|6666666.654935.peg.709	QLQ33790.1<br>QLQ33793.1
soxY	thiosulfate oxidation carrier protein SoxY (EC 1.8.2.6)	fig|1947697.4.peg.582<br>fig|1947697.4.peg.2162	QLQ33791.1<br>QLQ33794.1
soxB	thiosulfohydrolase SoxB (EC 3.1.6.20)	fig|6666666.654935.peg.2028	QLQ33787.1
soxX	sulfur oxidation c-type cytochrome SoxX (EC 1.8.2.3)	fig|6666666.654935.peg.3302<br>fig|6666666.654935.peg.572	QLQ32949.1<br>QLQ33795.1
soeA	sulfite dehydrogenase (quinone) subunit SoeA (EC 1.8.5.6)	fig|6666666.654935.peg.2056	WP_012971673.1<br>locus_tag="HZT40_19335"
soeB	sulfite dehydrogenase (quinone) subunit SoeB (EC 1.8.5.6)	fig|6666666.654935.peg.2057	QLQ34300.1
soeC	sulfite dehydrogenase (quinone) subunit SoeC (EC 1.8.5.6)	fig|6666666.654935.peg.2058	QLQ33396.1
aprA	adenylylsulfate reductase, subunit A (EC 1.8.4.10)	fig|6666666.654935.peg.3158	QLQ33717.1
aprB	adenylylsulfate reductase, subunit B (EC 1.8.4.10)	fig|6666666.654935.peg.3159	QLQ33716.1
sat	sulfate adenylyltransferase (EC 2.7.7.4)	fig|6666666.654935.peg.3160	QLQ33715.1
dsrA	Dissimilatory sulfite reductase, alpha subunit (EC 1.8.99.5)	fig|6666666.654935.peg.3292	QLQ32927.1
dsrB	Dissimilatory sulfite reductase, beta subunit (EC 1.8.99.5)	fig|6666666.654935.peg.3291	QLQ32926.1
dsrE	tRNA 2-thiouridine synthesizing protein D (EC 2.8.1.-)	fig|6666666.654935.peg.3290	QLQ32925.1
dsrF	tRNA 2-thiouridine synthesizing protein C (EC 2.8.1.-)	fig|6666666.654935.peg.3289	QLQ32924.1
dsrH	tRNA 2-thiouridine synthesizing protein B (EC 2.8.1.-)	fig|6666666.654935.peg.3288	QLQ32923.1
dsrC	tRNA 2-thiouridine synthesizing protein E (EC 2.8.1.-)	fig|6666666.654935.peg.3287<br>fig|6666666.654935.peg.3296	QLQ32922.1<br>QLQ30843.1
dsrM	sulfite reduction-associated complex DsrMKJOP protein DsrM	fig|6666666.654935.peg.3286	QLQ34178.1
dsrK	sulfite reduction-associated complex DsrMKJOP protein DsrK	fig|6666666.654935.peg.3285	QLQ32920.1
dsrL	glutamate synthase (NADPH) small chain dsrL/ intracellular sulfur oxidation protein DsrL	fig|6666666.654935.peg.3284	QLQ33191.1

dsrJ	Sulfite reduction-associated complex DsrMKJOP multiheme protein DsrJ	fig|6666666.654935.peg.3283	QLQ32918.1
dsrO	sulfite reduction-associated complex DsrMKJOP iron-sulfur protein DsrO	fig|6666666.654935.peg.3282	QLQ32917.1
dsrP	sulfite reduction-associated complex DsrMKJOP protein DsrP	fig|6666666.654935.peg.3281	QLQ32917.1
dsrN	Cobyrinic acid A,C-diamide synthase DsrN (EC 6.3.5.11)	fig|6666666.654935.peg.2759	QLQ31889.1
dsrR	protein involved in sulfur oxidation DsrR	fig|6666666.654935.peg.2760	—
dsrS	protein involved in sulfur oxidation DsrS	fig|6666666.654935.peg.40	QLQ31126.1
		**Nitrogen metabolism**	
narG	nitrate reductase alpha subunit (EC 1.7.5.1)	fig|6666666.654935.peg.651	QLQ32018.1
narH	nitrate reductase beta subunit (EC 1.7.5.1)	fig|6666666.654935.peg.652	QLQ32018.1
narI	nitrate reductase gamma subunit (EC 1.7.5.1)	fig|6666666.654935.peg.654	QLQ34178.1
narJ	nitrate reductase delta subunit (EC 1.7.5.1)	fig|6666666.654935.peg.653	WP_020558834.1 locus_tag="HZT40_10965"
nirS	nitrite reductase (NO-forming) (EC 1.7.2.1)	—	QLQ34181.1
cnorB	nitric oxide reductase subunit B (EC 1.7.2.5)	fig|6666666.654935.peg.1942	QLQ32042.1
cnorC	nitric oxide reductase subunit C (EC 1.7.2.5)	fig|6666666.654935.peg.1941	QLQ32043.1
nirB	nitrite reductase (NADH) large subunit (EC 1.7.1.1)	fig|6666666.654935.peg.1096	QLQ33725.1
nirD	nitrite reductase (NADH) small subunit (EC 1.7.1.1)	fig|6666666.654935.peg.1097	WP_002709036.1 locus_tag="HZT40_21300"
nifA	Nif-specific regulatory protein NifA	fig|6666666.654935.peg.634	QLQ31724.1
nifS	cysteine desulfurase NifS (EC 2.8.1.7)	fig|6666666.654935.peg.668	QLQ31672.1
nifU	nitrogen fixation protein NifU and related proteins	fig|6666666.654935.peg.667	QLQ30621.1
nifB	nitrogen fixation protein NifB	fig|6666666.654935.peg.656	QLQ31713.1
nifX	nitrogen fixation protein NifX	fig|6666666.654935.peg.2777	QLQ32343.1
nifX2	nitrogen fixation protein NifX2	fig|6666666.654935.peg.2779	QLQ32343.1
nifB2	nitrogen fixation protein NifB2	—	QLQ31713.1
nifY	nitrogen fixation-related protein, gamma subunit	fig|6666666.654935.peg.3527	—
nifE	nitrogenase molybdenum-cofactor synthesis protein NifE (EC 1.18.6.1)	fig|6666666.654935.peg.2774 fig|6666666.654935.peg.2775	QLQ34086.1
nNifN	nitrogenase molybdenum-iron protein NifN (EC 1.18.6.1.)	fig|6666666.654935.peg.2776	QLQ34087.1
nifQ	nitrogen fixation protein NifQ	fig|6666666.654935.peg.662	QLQ31677.1

nifV	homocitrate synthase NifV (EC 2.3.3.14)	fig|6666666.654935.peg.675	QLQ30738.1
nifW	nitrogen fixation protein NifW	fig|6666666.654935.peg.614	QLQ30752.1
nifM	nitrogen fixation protein NifM	fig|6666666.654935.peg.616	QLQ30754.1
nifH	nitrogenase iron protein NifH (EC 1.18.6.1)	fig|6666666.654935.peg.3522	QLQ32348.1
nifD	nitrogenase molybdenum-iron protein alpha chain (EC 1.18.6.1)	fig|6666666.654935.peg.3523	QLQ32347.1
nifK	nitrogenase molybdenum-iron protein beta chain (EC 1.18.6.1)	fig|6666666.654935.peg.3524	QLQ32346.1
nifZ	nitrogen fixation protein NifZ	fig|6666666.654935.peg.615	QLQ30753.1
nifT	nitrogen fixation protein NifT	fig|6666666.654935.peg.3525	QLQ32345.1
nifO	NifO	—	QLQ31710.1
		**Phosphorus metabolism**	
phoU	phosphate transport system protein PhoU	fig|6666666.654935.peg.2604	QLQ33978.1
phoR	Phosphate regulon sensor protein PhoR (EC 2.7.13.3)	fig|6666666.654935.peg.2602	QLQ32508.1
phoB	Phosphate regulon transcriptional regulatory protein PhoB (EC 2.7.13.3)	fig|6666666.654935.peg.1341<br>fig|6666666.654935.peg.2603	QLQ32507.1
pstS	phosphate transport system permease protein PstS	fig|6666666.654935.peg.1741<br>fig|6666666.654935.peg.260	QLQ31375.1
pstA	phosphate transport system permease protein PstA (EC 7.3.2.1)	fig|6666666.654935.peg.2606	QLQ31376.1<br>QLQ32506.1
pstC	phosphate transport system permease protein PstC (EC 7.3.2.1)	fig|6666666.654935.peg.2607	QLQ32505.1
pstB	pstA; phosphate transport system permease protein PstB (EC 7.3.2.1)	fig|6666666.654935.peg.2605	QLQ31377.1
ppk1	polyphosphate kinase (EC 2.7.4.1)	fig|6666666.654935.peg.1345	QLQ34227.1
epp	Exopolyphosphatase (EC 3.6.1.11)	fig|6666666.654935.peg.540	QLQ33092.1
		**Embden-Meyerhof (EM) pathway and Calvin-Benson-Bassham cycle**	
glk	Glucokinase (EC 2.7.1.2)	fig|6666666.654935.peg.695<br>fig|6666666.654935.peg.2090	QLQ32326.1
pgi	glucose-6-phosphate isomerase (EC 5.3.1.9)	fig|6666666.654935.peg.2089	QLQ33478.1
pfk	6-phosphofructokinase (EC 2.7.1.11)	fig|6666666.654935.peg.3154	QLQ33720.1
fbaA	fructose-bisphosphate aldolase class II (EC 4.1.2.13)	fig|6666666.654935.peg.2989	QLQ33878.1
tpiA	triosephosphate isomerase (EC 5.3.1.1)	fig|6666666.654935.peg.1194	QLQ32910.1
gapA	NAD-dependent glyceraldehyde-3-phosphate dehydrogenase (EC 1.2.1.12)	fig|6666666.654935.peg.1302<br>fig|6666666.654935.peg.2986	QLQ33881.1
pgk	Phosphoglycerate kinase (EC 2.7.2.3)	fig|6666666.654935.peg.2987	QLQ33880.1

gpmI	2,3-bisphosphoglycerate-independent phosphoglycerate mutase (EC 5.4.2.1)	fig|6666666.654935.peg.974	QLQ34313.1
gpmB	alpha-ribazole-5'-phosphate phosphatase (EC 3.1.3.73)	fig|6666666.654935.peg.1632	—
eno	Enolase (EC 4.2.1.11)	fig|6666666.654935.peg.2493	QLQ31148.1
pyk	Pyruvate kinase (EC 2.7.1.40)	fig|6666666.654935.peg.2988	QLQ33879.1<br>QLQ31383.1
aceE	Pyruvate dehydrogenase E1 component (EC 1.2.4.1)	fig|6666666.654935.peg.745<br>fig|6666666.654935.peg.2843	QLQ33197.1<br>QLQ31452.1
pdhC	Dihydrolipoamide acetyltransferase component of pyruvate dehydrogenase complex (EC 2.3.1.12)	fig|6666666.654935.peg.746<br>fig|6666666.654935.peg.2844	QLQ33196.1
pdhD	Dihydrolipoamide dehydrogenase of pyruvate dehydrogenase complex (EC 1.8.1.4)	fig|6666666.654935.peg.747<br>fig|6666666.654935.peg.1364<br>fig|6666666.654935.peg.2845	QLQ33195.1<br>QLQ31603.1<br>WP_019023766.1<br>locus_tag="HZT40_07365"
rpiA	Ribose 5-phosphate isomerase A (EC 5.3.1.6)	fig|6666666.654935.peg.1410	QLQ30286.1
rpe	Ribulose-phosphate 3-epimerase (EC 5.1.3.1)	—	QLQ31009.1
tktA	Transketolase (EC 2.2.1.1)	fig|6666666.654935.peg.2985	QLQ33882.1
fbp	Fructose-1,6-bisphosphatase, type I (EC 3.1.3.11)	fig|6666666.654935.peg.1039	present, but not annotated
rbcL	Ribulose bisphosphate carboxylase large chain (EC 4.1.1.39)	fig|6666666.654935.peg.1163<br>fig|6666666.654935.peg.900	QLQ31332.1
rbcS	Ribulose bisphosphate carboxylase small chain (EC 4.1.1.39)	fig|6666666.654935.peg.901<br>fig|6666666.654935.peg.1164	QLQ31406.1
prkB	Phosphoribulokinase (EC 2.7.1.19)	fig|6666666.654935.peg.948	QLQ33508.1
**Tricarboxylic acid (TCA) cycle, glyoxylate cycle and a way to anabolism from TCA**			
gltA	Citrate synthase (si) (EC 2.3.3.1)	fig|6666666.654935.peg.1367	QLQ31602.1
acnB	Aconitate hydratase 2 (EC 4.2.1.3)	fig|6666666.654935.peg.1368	QLQ31601.1
idh	Isocitrate dehydrogenase (NADP) (EC 1.1.1.42)	fig|6666666.654935.peg.2560<br>fig|6666666.654935.peg.1253	QLQ31348.1<br>QLQ31637.1
sucA	2-oxoglutarate dehydrogenase E1 component (EC 1.2.4.2)	fig|6666666.654935.peg.1361<br>fig|6666666.654935.peg.2190	QLQ31177.1<br>QLQ31079.1
sucB	2-oxoglutarate dehydrogenase E2 complex (EC 2.3.1.61)	fig|6666666.654935.peg.1363	QLQ31604.1
sucD	Succinyl-CoA ligase (ADP-forming) alpha chain (EC 6.2.1.5)	fig|6666666.654935.peg.1366	WP_002706977.1<br>locus_tag="HZT40_08370"
sucC	Succinyl-CoA ligase (ADP-forming) beta chain (EC 6.2.1.5)	fig|6666666.654935.peg.1365	WP_002706976.1<br>locus_tag="HZT40_08375"
sdhA	Succinate dehydrogenase flavoprotein subunit (EC 1.3.99.1)	fig|6666666.654935.peg.1357	QLQ31174.1

sdhB	Succinate dehydrogenase iron-sulfur protein (EC 1.3.99.1)	fig|6666666.654935.peg.1356	QLQ31173.1
sdhC	Succinate dehydrogenase cytochrome b-556 subunit (EC 1.3.99.1)	fig|6666666.654935.peg.1359	QLQ31175.1
sdhD	Succinate dehydrogenase hydrophobic membrane anchor protein (EC 1.3.5.1)	fig|6666666.654935.peg.1358	WP_002706930.1 locus_tag="HZT40_05675"
fumA	Fumarate hydratase class I, aerobic (EC 4.2.1.2)	fig|6666666.654935.peg.2555	QLQ31155.1
fumC	Fumarate hydratase class I, aerobic (EC 4.2.1.2)	—	QLQ31685.1
mqo	Malate:quinone oxidoreductase (EC 1.1.5.4)	fig|6666666.654935.peg.2675	QLQ32494.1
pckA	Phosphoenolpyruvate carboxykinase (ATP) (EC 4.1.1.49)	fig|6666666.654935.peg.3025	QLQ31415.1
aspB	Aspartate aminotransferase (EC 2.6.1.1)	fig|6666666.654935.peg.2488	QLQ34144.1
gltB	Glutamate synthase (NADPH) large chain (EC 1.4.1.13)	—	QLQ32096.1
gltD	Glutamate synthase (NADPH) small chain (EC 1.4.1.13)	fig|6666666.654935.peg.751	QLQ32571.1 WP_010871407.1 locus_tag="HZT40_11410"
aceA	Isocitrate lyase (EC 4.1.3.1)	fig|6666666.654935.peg.2674	WP_002554010.1 locus_tag="HZT40_13880"
aceB	Malate synthase G (EC 2.3.3.9)	fig|6666666.654935.peg.2950	QLQ33085.1
maeB	NADP-dependent malic enzyme (EC 1.1.1.40)	fig|6666666.654935.peg.1819 fig|6666666.654935.peg.2001	QLQ33532.1 QLQ33315.1
ppc	phosphoenolpyruvate carboxylase (EC 4.1.1.31)	—	QLQ32413.1
pyc	Pyruvate carboxylase (EC 6.4.1.1)	fig|6666666.654935.peg.1183	QLQ31324.1
	**Antioxidant protection**		
SOD2	Superoxide dismutase [Fe] (EC 1.15.1.1)	fig|6666666.654935.peg.134	QLQ30489.1
CAT2	catalase/peroxidase HPI (EC 1.11.1.21)	—	QLQ32619.1
	**Respiratory Electron Transfer Chain**		
	**Complex 1 NADH-dehydrogenase**		
nuoA	NADH ubiquinone oxidoreductase chain A (EC 1.6.5.3)	fig|6666666.654935.peg.1196	WP_012982121.1 locus_tag="HZT40_16395"
nouB	NADH-ubiquinone oxidoreductase chain B (EC 1.6.5.3)	fig|6666666.654935.peg.1197	QLQ32569.1 WP_004630808.1 locus_tag="HZT40_16390"
nuoC	NADH-ubiquinone oxidoreductase chain C (EC 1.6.5.3)	fig|6666666.654935.peg.1198	QLQ32570.1 QLQ32909.1
nuoD	NADH-ubiquinone oxidoreductase chain D (EC 1.6.5.3)	fig|6666666.654935.peg.1199	QLQ32908.1 WP_002707159.1 locus_tag="HZT40_14375"
nuoE	NADH-ubiquinone oxidoreductase chain E (EC 1.6.5.3)	fig|6666666.654935.peg.1200	QLQ34262.1

nuoF	NADH-ubiquinone oxidoreductase chain F (EC 1.6.5.3)	fig|6666666.654935.peg.1201	QLQ32907.1<br>QLQ32567.1
nuoG	NADH-ubiquinone oxidoreductase chain G (EC 1.6.5.3)	fig|6666666.654935.peg.1202	QLQ32906.1
nuoH	NADH-ubiquinone oxidoreductase chain H (EC 1.6.5.3)	fig|6666666.654935.peg.1203	QLQ32905.1<br>QLQ32568.1
nuoI	NADH-ubiquinone oxidoreductase chain I (EC 1.6.5.3)	fig|6666666.654935.peg.1204	QLQ32904.1
nuoJ	NADH-ubiquinone oxidoreductase chain J (EC 1.6.5.3)	fig|6666666.654935.peg.1205	QLQ32903.1
nuoK	NADH-ubiquinone oxidoreductase chain K (EC 1.6.5.3)	fig|6666666.654935.peg.1206	QLQ32902.1<br>QLQ32566.1
nuoL	NADH-ubiquinone oxidoreductase chain L (EC 1.6.5.3)	fig|6666666.654935.peg.1207	QLQ32901.1<br>QLQ33633.1
nuoM	NADH-ubiquinone oxidoreductase chain M (EC 1.6.5.3)	fig|6666666.654935.peg.1208	QLQ33636.1<br>QLQ33632.1<br>WP_008291478.1<br>locus_tag="HZT40_16335"
nuoN	NADH-ubiquinone oxidoreductase chain N (EC 1.6.5.3)	fig|6666666.654935.peg.1209	QLQ32900.1
**Complex II (succinate dehydrogenase)**			
sdhC	Succinate dehydrogenase, cytochrome b556 subunit (EC 1.3.99.1)	fig|6666666.654935.peg.1359	QLQ31175.1
sdhD	Succinate dehydrogenase, hydrophobic membrane anchor protein (EC 1.3.5.1)	fig|6666666.654935.peg.1358	WP_002706930.1<br>locus_tag="HZT40_05675"
sdhA	Succinate dehydrogenase flavoprotein subunit (EC 1.3.99.1)	fig|6666666.654935.peg.1357	QLQ31174.1
sdhB	Succinate dehydrogenase iron-sulfur protein (EC 1.3.99.1)	fig|6666666.654935.peg.1356	QLQ31173.1
**Complex III**			
cytB	ubiquinol-cytochrome c reductase cytochrome b subunit (EC 7.1.1.8)	fig|6666666.654935.peg.1086<br>fig|6666666.654935.peg.277	QLQ31660.1
cyc1	ubiquinol-cytochrome c reductase cytochrome c1 subunit (EC 7.1.1.8)	fig|6666666.654935.peg.1085	QLQ31659.1
rip1 (ISP)	ubiquinol-cytochrome c reductase iron-sulfur subunit (EC 7.1.1.8)	fig|6666666.654935.peg.1087	QLQ31661.1<br>QLQ31570.1
**Complex IV (terminal oxydases)**			
cyoE	Heme A synthase, cytochrome oxidase biogenesis protein Cox15-CtaA (EC 2.5.1.141)	fig|6666666.654935.peg.115	QLQ31913.1
cox11	cytochrome c oxidase assembly protein subunit 11 (EC 7.1.1.9)	fig|6666666.654935.peg.109	QLQ31908.1<br>QLQ31900.1

cox15	cytochrome c oxidase assembly protein subunit 15 (EC 7.1.1.9)	fig|6666666.654935.peg.1879	QLQ31913.1
coxB	cytochrome c oxidase subunit II (EC 7.1.1.9)	fig|6666666.654935.peg.106	QLQ31902.1<br>QLQ31910.1
coxA	cytochrome c oxidase subunit I (EC 7.1.1.9)	fig|6666666.654935.peg.107	QLQ31901.1<br>QLQ31909.1
coxC	cytochrome c oxidase subunit III (EC 7.1.1.9)	fig|6666666.654935.peg.110	QLQ31907.1<br>QLQ31899.1<br>QLQ34175.1
coxD	cytochrome c oxidase subunit IV (EC 7.1.1.9)		QLQ32013.1
cydA	Cytochrome d ubiquinol oxidase subunit I (EC 7.1.1.7)	fig|6666666.654935.peg.2173	QLQ31581.1
cydB	Cytochrome d ubiquinol oxidase subunit II (EC 7.1.1.7)	fig|6666666.654935.peg.2172	QLQ31580.1
cydC	Transport ATP-binding protein CydC	fig|6666666.659398.peg.1687<br>fig|6666666.659398.peg.1690	WP_002706844.1<br>locus_tag="HZT40_08230"
cydD	Transport ATP-binding protein CydD	fig|6666666.654935.peg.2171	QLQ31578.1
cydX	cytochrome bd-I ubiquinol oxidase subunit X (EC 7.1.1.7)		QLQ31579.1
ccoN	cytochrome c oxidase cbb3-type subunit I (EC 1.9.3.1)	fig|6666666.654935.peg.811<br>fig|6666666.654935.peg.1292	QLQ32538.1<br>QLQ30879.1<br>QLQ32042.1<br>locus_tag="HZT40_10230"
ccoO	cytochrome c oxidase cbb3-type subunit II (EC 7.1.1.9)	fig|6666666.654935.peg.810<br>fig|6666666.654935.peg.1293	QLQ30880.1<br>QLQ32537.1
ccoP	cytochrome c oxidase cbb3-type subunit III (EC 7.1.1.9)	fig|6666666.654935.peg.808<br>fig|6666666.654935.peg.1295	QLQ30881.1<br>QLQ34081.1<br>QLQ32536.1
ccoG (FixG)	Type cbb3 cytochrome oxidase biogenesis protein CcoG, involved in Cu oxidation	fig|6666666.659398.peg.1742<br>fig|6666666.659398.peg.2266<br>fig|6666666.659398.peg.805	QLQ30882.1<br>QLQ31613.1
ccoQ	CcoQ/FixQ family Cbb3-type cytochrome c oxidase assembly chaperone (EC 1.9.3.1)	fig|6666666.659398.peg.803	QLQ34234.1
ccoS	cbb3-type cytochrome oxidase assembly protein CcoS	fig|6666666.659398.peg.1739	WP_020559959.1<br>locus_tag="HZT40_08450"
	**ATPase**		
atpA	F-type H+-transporting ATPase subunit alpha (EC 7.1.2.2)	fig|6666666.654935.peg.180	QLQ32745.1
atpD	F-type H+-transporting ATPase subunit beta (EC 7.1.2.2)	fig|6666666.654935.peg.178	QLQ32743.1
atpH	F-type H+-transporting ATPase subunit delta (EC 7.1.2.2)	fig|6666666.654935.peg.181	QLQ32746.1
atpC	F-type H+-transporting ATPase subunit epsilon (EC 7.1.2.2)	fig|6666666.654935.peg.177	QLQ32742.1
atpG	F-type H+-transporting ATPase subunit gamma (EC 7.1.2.2)	fig|6666666.654935.peg.179	QLQ32744.1

atpB	F-type H+-transporting ATPase subunit a (EC 7.1.2.2)	fig|6666666.654935.peg.184	QLQ32712.1<br>QLQ32748.1
atpF	F-type H+-transporting ATPase subunit b (EC 7.1.2.2)	fig|6666666.654935.peg.182	QLQ32747.1
atpE	ATP synthase F0 sector subunit c (EC 7.1.2.2)	fig|6666666.654935.peg.183	QLQ34252.1
